# Supplementary figures and images for: The tRNA epitranscriptomic landscape and RNA modification enzymes in Vibrio cholerae
Source: PLoS Genet. 2025 Oct 31;21(10):e1011937. doi: 10.1371/journal.pgen.1011937 (PMC12588516; doi:10.1371/journal.pgen.1011937)

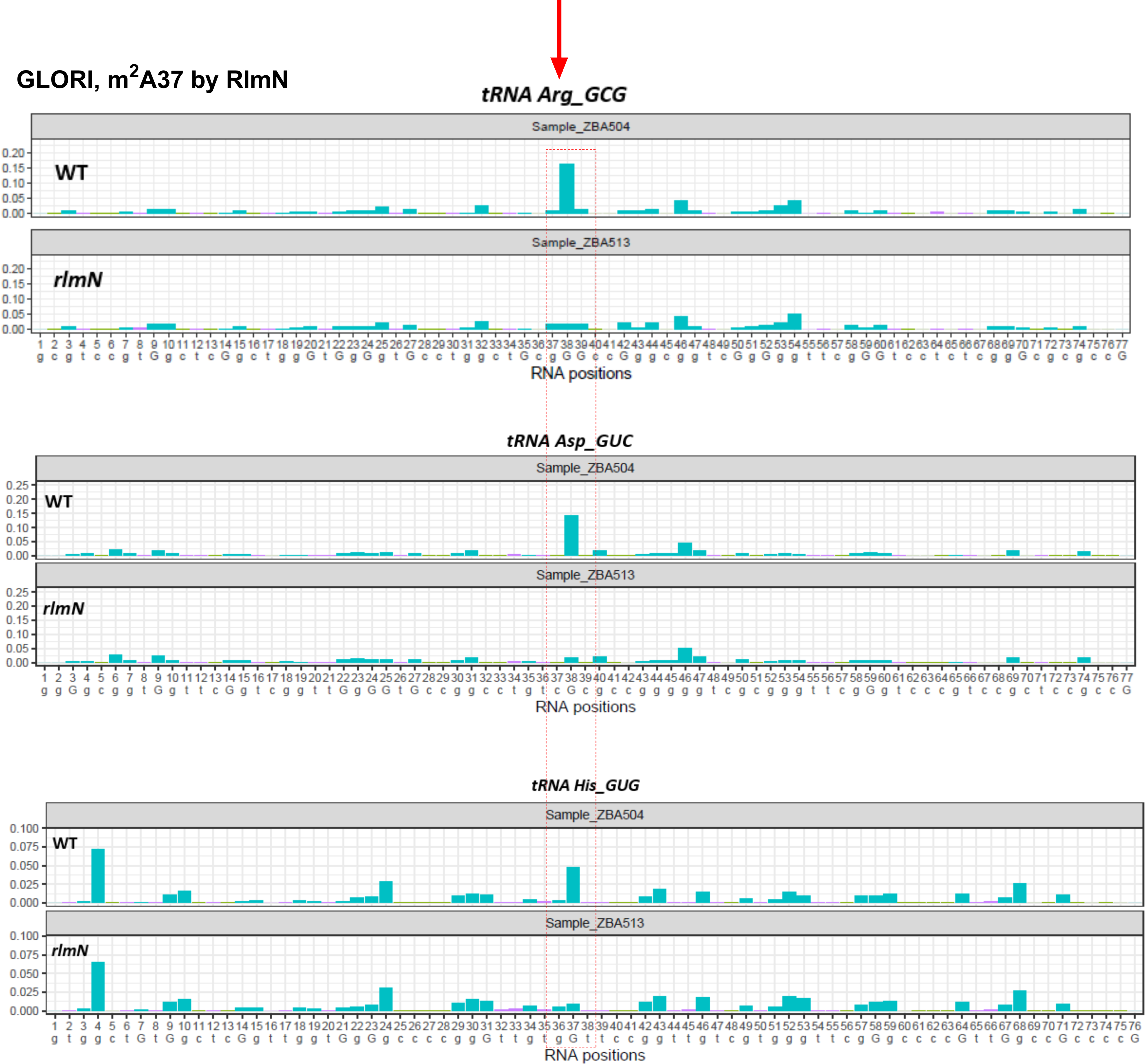

Supplement: S1 Fig — Histograms show the A-to-G deamination rate (y-axis) at the indicated nucleotide positions (x-axis). A → G substitutions indicate unmodified adenosines, whereas retention of A signal (GtoA score, see legend to the Fig 1) denotes the presence of a modification. Note that the RlmN-dependent signal was weak and detected only in the three tRNAs shown. (TIF) [file pgen.1011937.s001.tif]

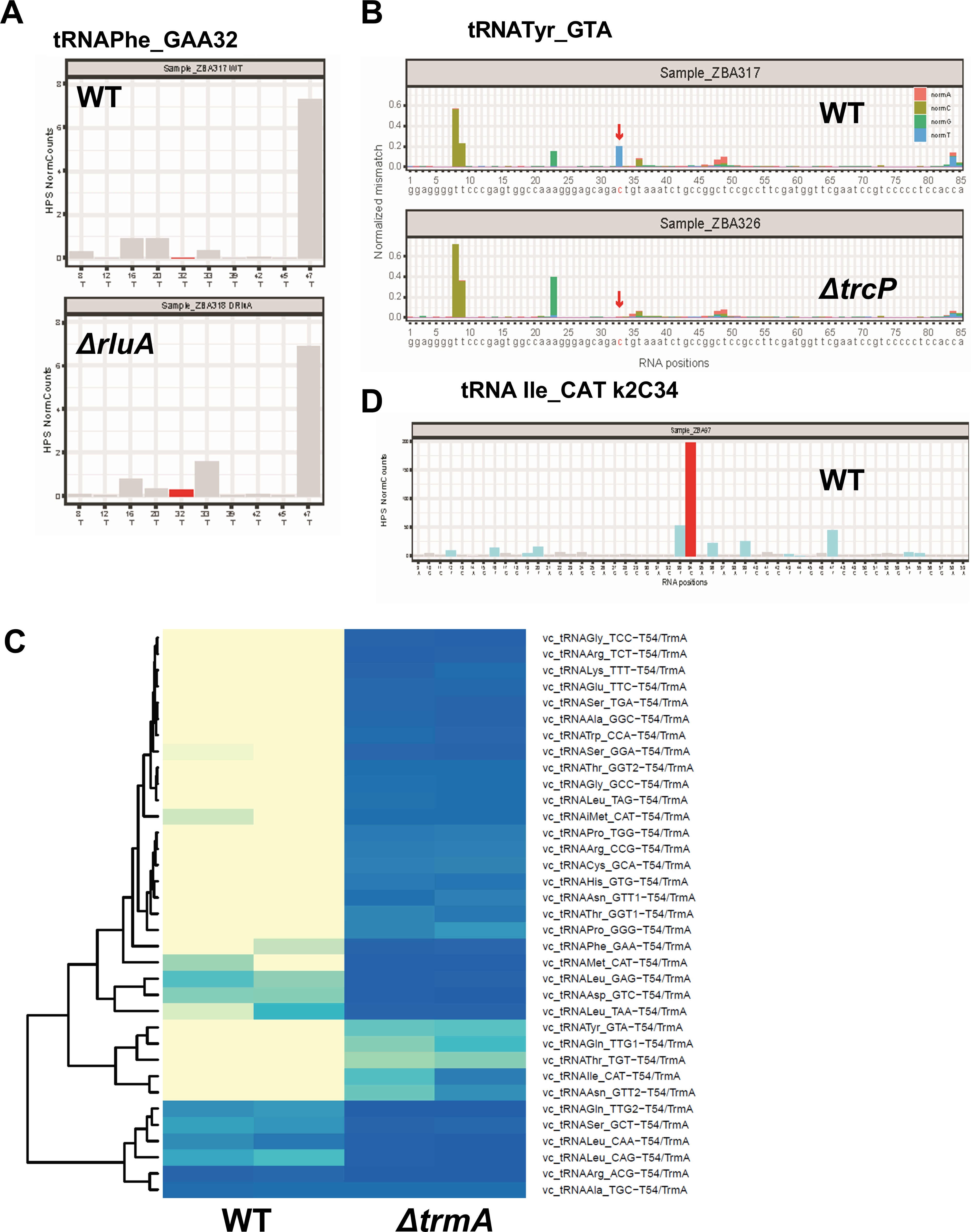

Supplement: S2 Fig — AB: Detection of Ψ32 made by RluA (A) and C to U conversion by TrcP, followed by conversion to Ψ32 (B). HydraPsiSeq uses hydrazine to cleave unmodified uridines, with Ψ residues resisting cleavage and producing a protection signal. With BID-Seq, Ψ positions show up as characteristic single-base deletions (or stops) compared to the reference. C: Heatmap of m5U (T54) modifications detected by HydraPsiSeqin WT, and disappearance in ∆trmA. Blue indicates absence of modification while yellow means presence D: Detection of k2C34 in WT as cleavage at C34 residue in tRNAIle_CAT. (TIF) [file pgen.1011937.s002.tif]

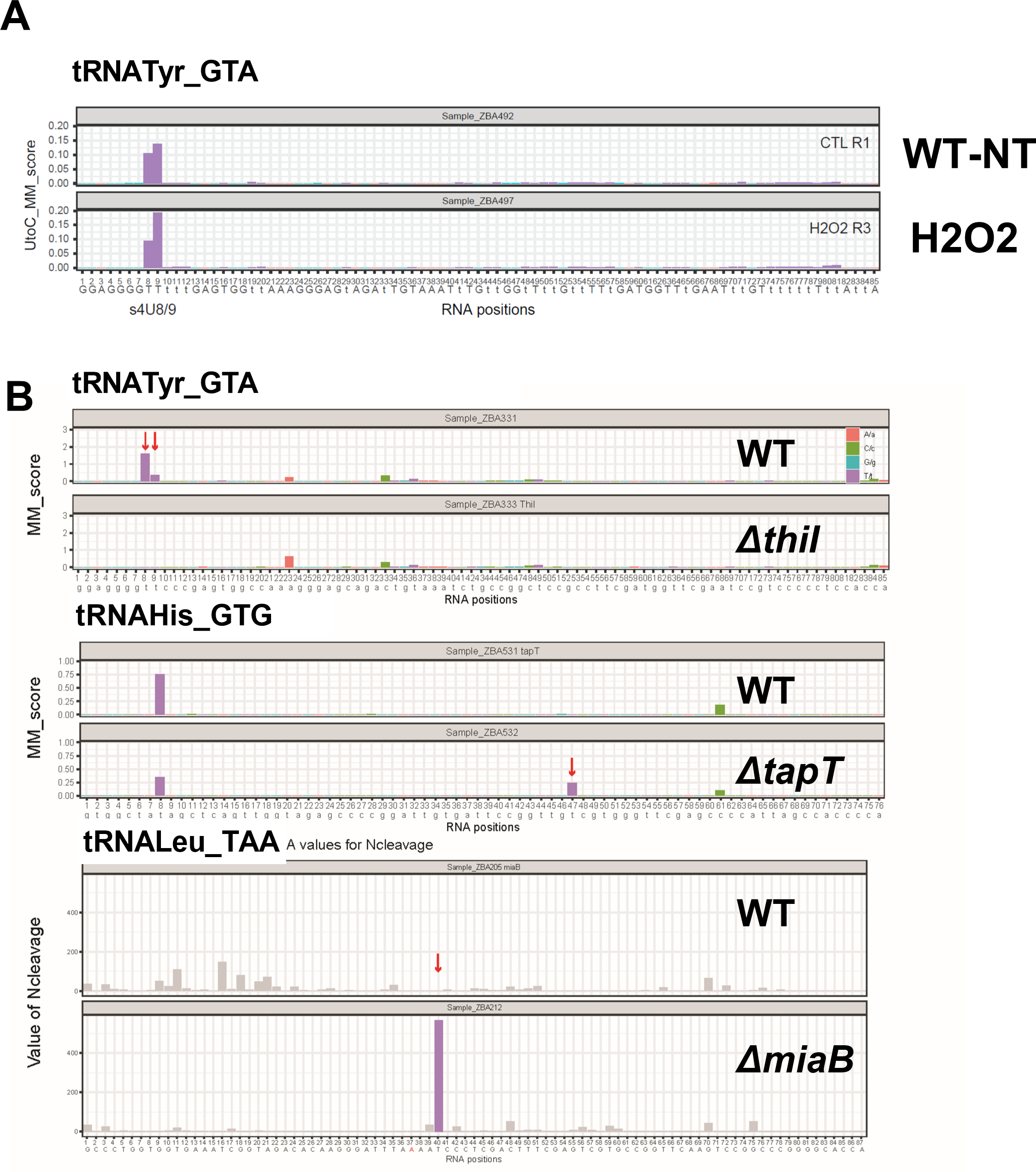

Supplement: S3 Fig — B. Analysis of RT-signatures for mapping of RT-mismatching and RT-arresting tRNA modifications. Detection of s4U at positions 8 and 9 in WT and the absence of the signal in the deletion mutant for ∆thiI. acp3U47 detected in WT but not in ∆tapT. ms2i6A37 signal detected in WT but not in ∆miaB. (TIF) [file pgen.1011937.s003.tif]

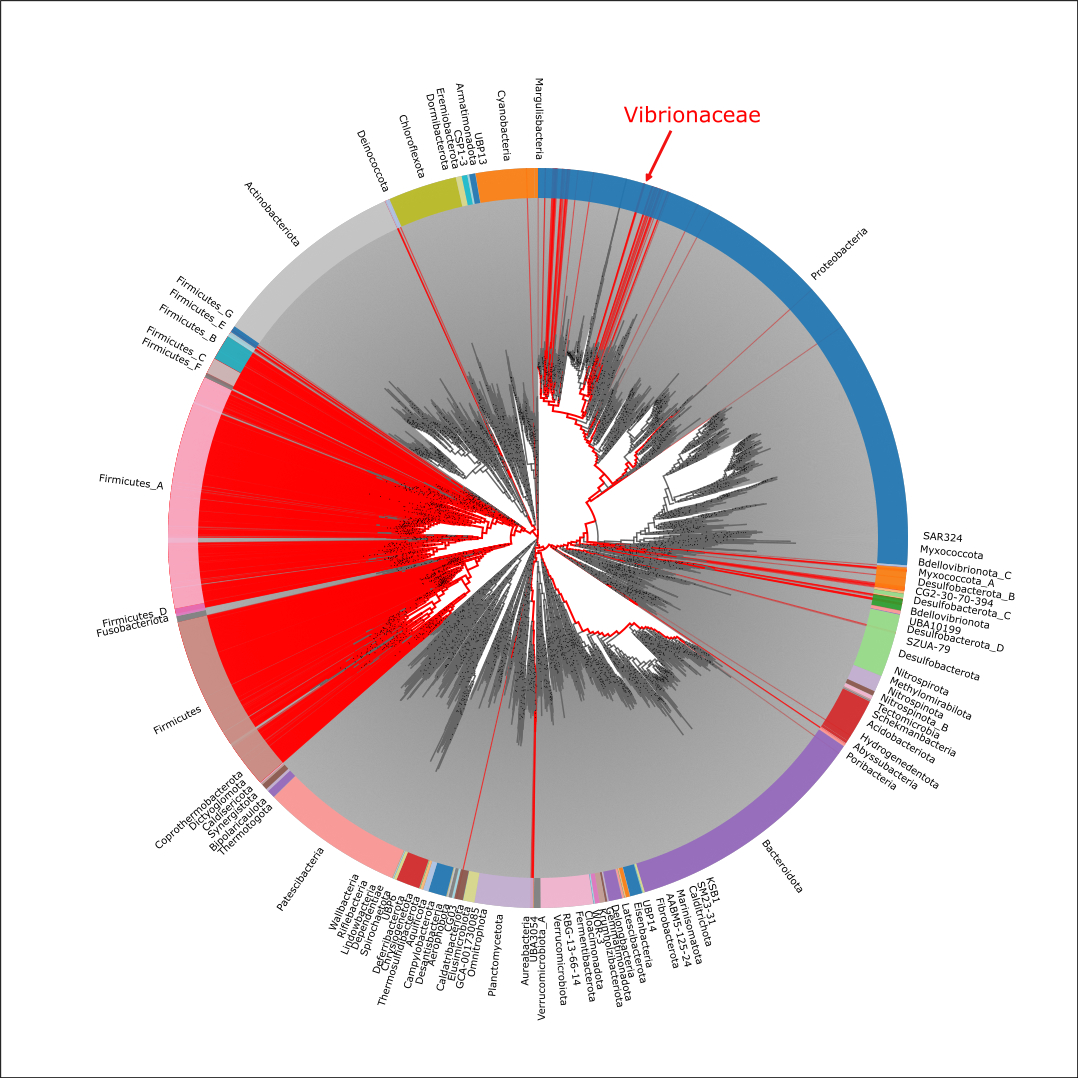

Supplement: S4 Fig — AnnoTree visualizes annotations across a large phylogenetic tree, here comprising 80,000 bacterial genomes (one genome per species). trmK was found in 17,363 genomes. Absence (grey) or presence (colored) of an annotated trmK homolog is shown for each genome. The position of the Vibrionaceae family is indicated with a red arrow. Each phylum name is color-coded. (TIF) [file pgen.1011937.s004.tif]

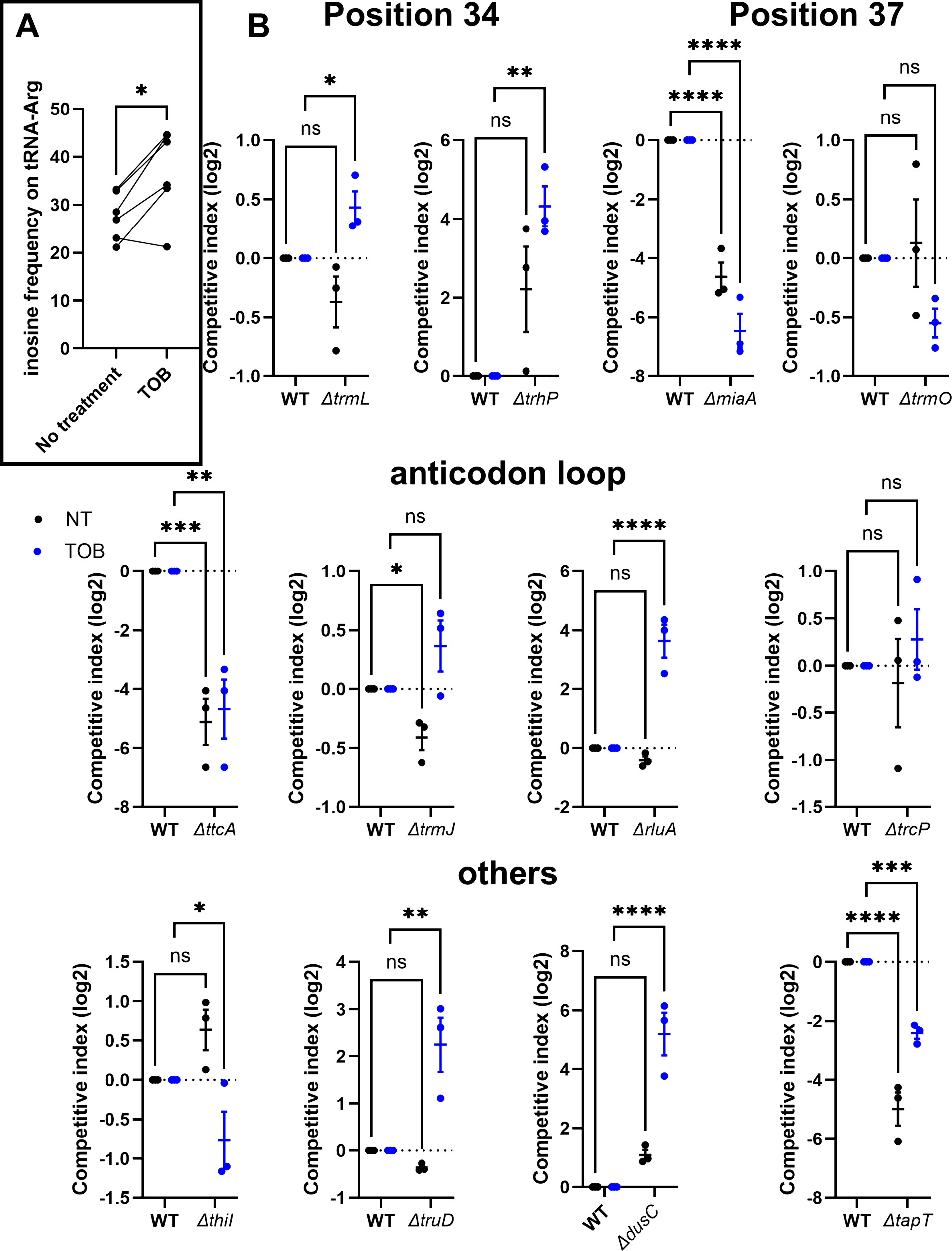

Supplement: S5 Fig — A. Inosine frequency on tRNAArg detected in RNA-seq performed on V. cholerae without and with subinhibitory concentration of tobramycin (TOB, 0.4 µg/ml). For statistical analysis, student’s t-test was used. * means p < 0.05. B. In vitro competition experiments of V. cholerae WT and mutant strains in the absence or presence of tobramycin at sub-MICs (50% of the MIC): 0.6 μg/ml. y-axis: log2 of competitive index value of the tested strain against the WT, calculated as described in the methods. Values shown for WT are competitions between 2 isogenic lacZ + vs lacZ- WT strains. A competitive index of 1 indicates equal growth of both strains. NT: no antibiotic treatment. For multiple comparisons, we used one-way ANOVA **** means P < .0001, *** means P < .001, ** means P < .01, and * means P < .05. Only significant P-values are represented. Number of biological replicates for each experiment: n = 3. (TIF) [file pgen.1011937.s005.tif]

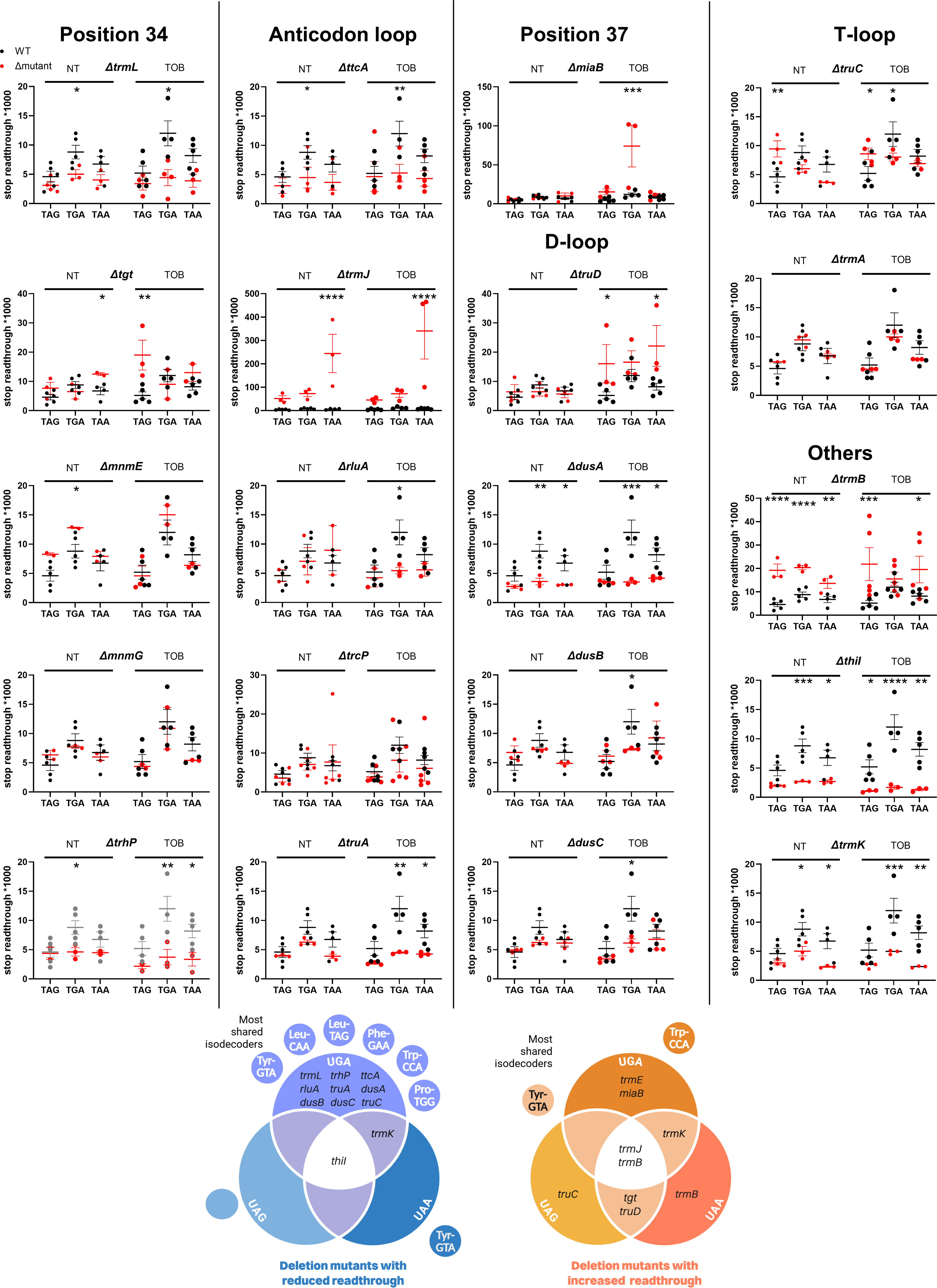

Supplement: S6 Fig — Black: wild-type (WT). Red: ∆mutant. NT: no treatment. TOB: growth in the presence of tobramycin at 20% of the MIC. Reporters described in Fabret and Namy, 2021, and Fruchard et al 2025. Y-axis represents stop codon readthrough*1000. Number of biological replicates: between 3 and 6. For multiple comparisons, we used one-way ANOVA. **** means p < 0.0001, *** means p < 0.001, ** means p < 0.01, * means p < 0.05. Venn diagrams showing overlap of tRNA-modification enzyme deletion mutants that (orange) increase or (blue) decrease stop-codon readthrough across UAA, UAG, and UGA. Small circles indicate the most shared tRNA isodecoders among enzymes in each group; for instance, UAA readthrough is reduced in thiI and trmK mutants, both targeting tRNATyr_GTA. (TIF) [file pgen.1011937.s006.tif]

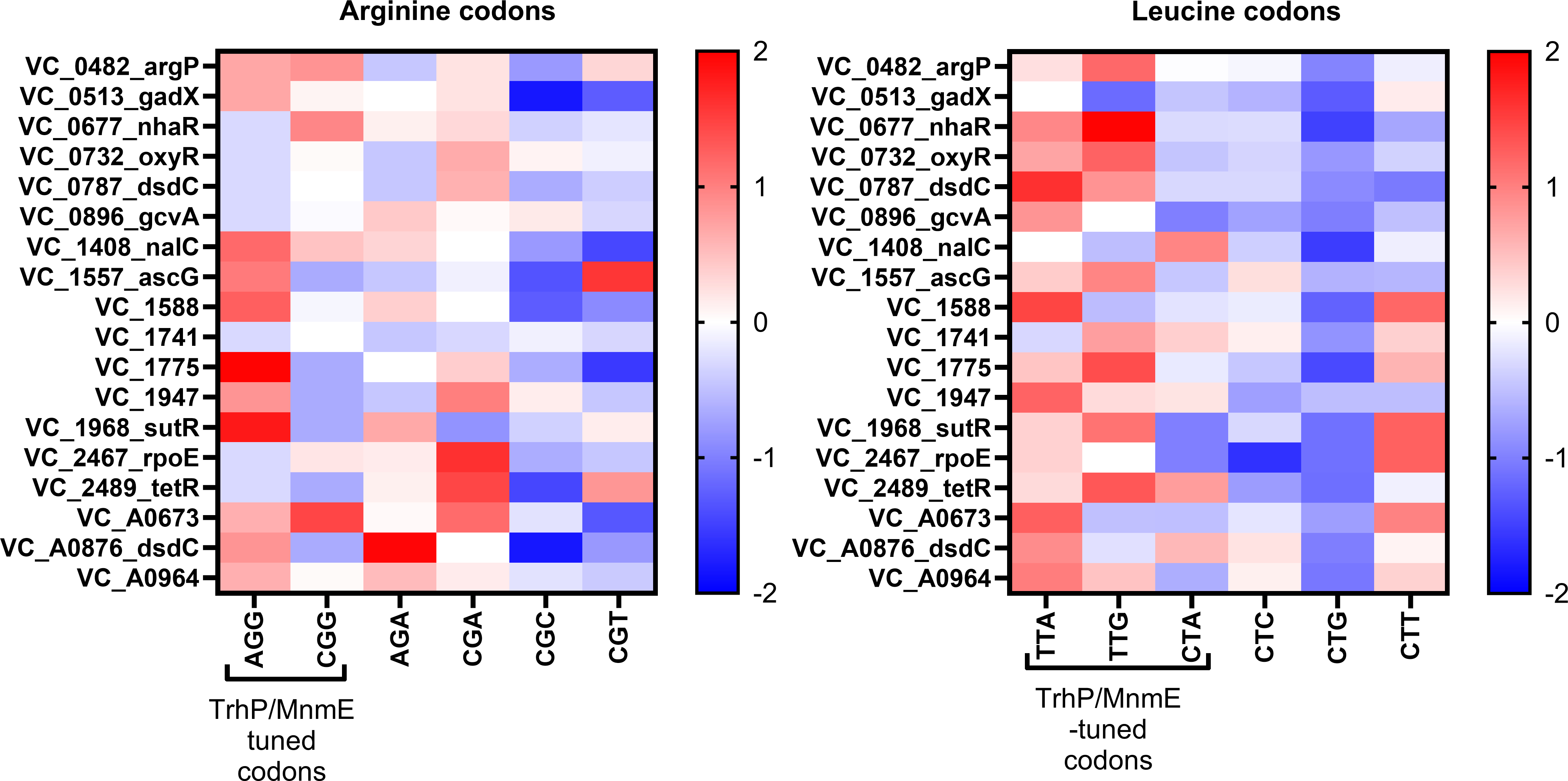

Supplement: S7 Fig — Standardized codon usage bias is shown for the codons of interest. In the plots, red indicates a positive codon usage bias (the codon occurs more frequently than expected relative to the genome-wide mean), whereas blue indicates a negative codon usage bias (the codon is underrepresented compared with the genomic average). (TIF) [file pgen.1011937.s007.tif]

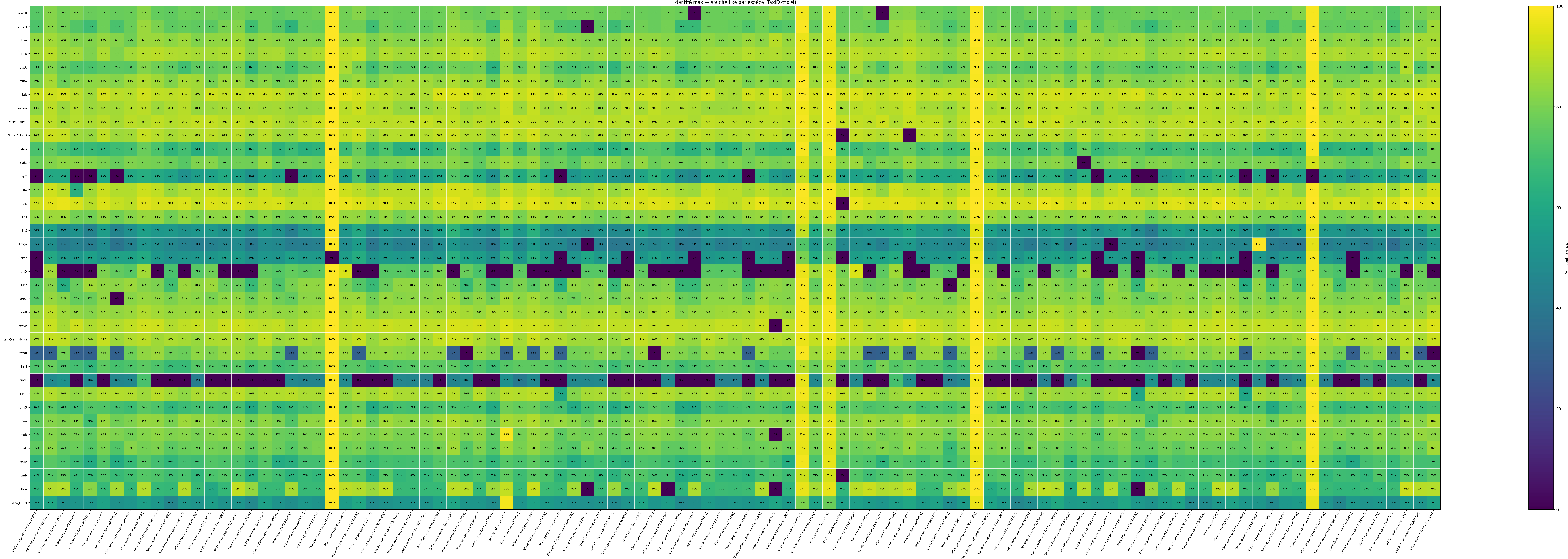

Supplement: S8 Fig — Heatmap showing the presence and sequence identity of tRNA-modification enzymes (rows) across Vibrio species (columns). Color intensity represents amino acid sequence identity relative to the Vibrio cholerae N16961 reference, with yellow indicating 100% identity and shades toward purple indicating lower identity. Purple squares denote complete absence of the corresponding gene. All V. cholerae strains show full presence of the enzymes. (TIF) [file pgen.1011937.s008.tif]
